# The role and therapeutic potential of nanotechnology-mediated ferroptosis regulation in myelodysplastic syndromes

**DOI:** 10.3389/fonc.2026.1887533

**Published:** 2026-07-01

**Authors:** Wanhua An, Shuli Guo, Haojie Wang, Tao Lv, Wanli Wang, Bo Li, Mengyu Liu, Pu Yang, Farra Aidah Jumuddin

**Affiliations:** 1 School of Medicine, Lincoln University College, Petaling Jaya, Malaysia; 2 Department of Hematology, Luoyang Central Hospital Affiliated to Zhengzhou University, Luoyang, Henan, China; 3 Central Laboratory, Luoyang Central Hospital Affiliated to Zhengzhou University, Luoyang, Henan, China

**Keywords:** ferroptosis, iron overload, microenvironment, myelodysplastic syndrome, nanotechnology

## Abstract

Myelodysplastic syndromes (MDS) are a heterogeneous group of clonal hematopoietic stem cell disorders characterized by ineffective hematopoiesis and an increased risk of progression to acute myeloid leukemia (AML). Patients with MDS often develop progressive systemic iron overload due to ineffective erythropoiesis, dysregulated hepcidin expression, and repeated blood transfusions. Excess free iron promotes reactive oxygen species generation through the Fenton reaction, resulting in persistent oxidative stress within the bone marrow microenvironment. These abnormalities are closely associated with ineffective hematopoiesis. Ferroptosis is an iron-dependent form of regulated cell death driven by lipid peroxidation. In MDS, ferroptosis appears to play a dual role. In lower-risk disease settings characterized by ineffective erythropoiesis and anemia, ferroptosis-related injury in erythroid-lineage cells may exacerbate ineffective erythropoiesis. In contrast, in higher-risk or clonally progressive disease settings, selective induction of ferroptosis in malignant clonal cells may represent a potential therapeutic strategy. However, current ferroptosis-targeting agents still face several limitations, including poor water solubility, low stability *in vivo*, insufficient bone marrow targeting, and possible toxicity to normal tissues. Nanotechnology may offer a promising approach to address these challenges. Nanocarriers can improve bone marrow targeting through surface modification and optimized physicochemical properties, while also enabling controlled drug release and co-delivery of multiple therapeutic agents to enhance efficacy and reduce side effects. This review summarizes the role of ferroptosis in the pathogenesis and treatment of MDS, discusses the major challenges in current ferroptosis-based therapies, and highlights the opportunities and challenges of nanotechnology-mediated ferroptosis regulation as a potential future strategy for precision treatment of MDS.

## 1 Introduction

MDS is a heterogeneous myeloid neoplasm originating from hematopoietic stem and progenitor cells, characterized by ineffective hematopoiesis, peripheral cytopenia, and a high risk of leukemic transformation (1). Clinical outcomes vary significantly across different risk groups. Lower-risk patients may survive for decades, whereas higher-risk patients experience rapid disease progression, with some potentially dying within months or even weeks (2). Based on the Revised International Prognostic Score System (IPSS‐R) for MDS, patients with an IPSS‐R score ≤ 3.5 were categorised as the low‐risk (LR) group, while those with an IPSS‐R score > 3.5 were designated as the high‐risk (HR) group (3–5). In recent years, the application of treatments such as hypomethylating agents (HMAs) has improved outcomes in some patients, but drug resistance and relapse remain prominent issues (6). Therefore, exploring new and more targeted treatment strategies is an important direction for current MDS research.

Iron metabolism dysregulation is one of the key biological features of MDS (7–9). Ineffective hematopoiesis and repeated blood transfusions can lead to systemic iron overload, while excess free Fe²^+^ promotes reactive oxygen species generation and lipid peroxidation through the Fenton reaction (10, 11). These abnormalities create a biological context favorable for ferroptosis, an iron-dependent form of regulated cell death driven by lipid peroxidation (12, 13). In MDS, ferroptosis is particularly relevant because chronic iron overload and oxidative stress are closely associated with disease development, ineffective hematopoiesis, and treatment responses (14–17). Current evidence suggests that ferroptosis may exert distinct effects across different biological and clinical contexts of MDS. In lower-risk disease settings characterized by ineffective erythropoiesis and anemia, iron overload and oxidative stress may promote ferroptosis-related injury in erythroid cells, thereby worsening ineffective hematopoiesis (17, 18). In contrast, in higher-risk or clonally progressive disease settings, selective induction of ferroptosis in malignant cells may represent a potential therapeutic strategy (19, 20). This context-dependent dual role highlights ferroptosis as both a pathogenic mechanism and a potential therapeutic target in MDS. However, current ferroptosis inducers still have several limitations.

**Figure 1:**
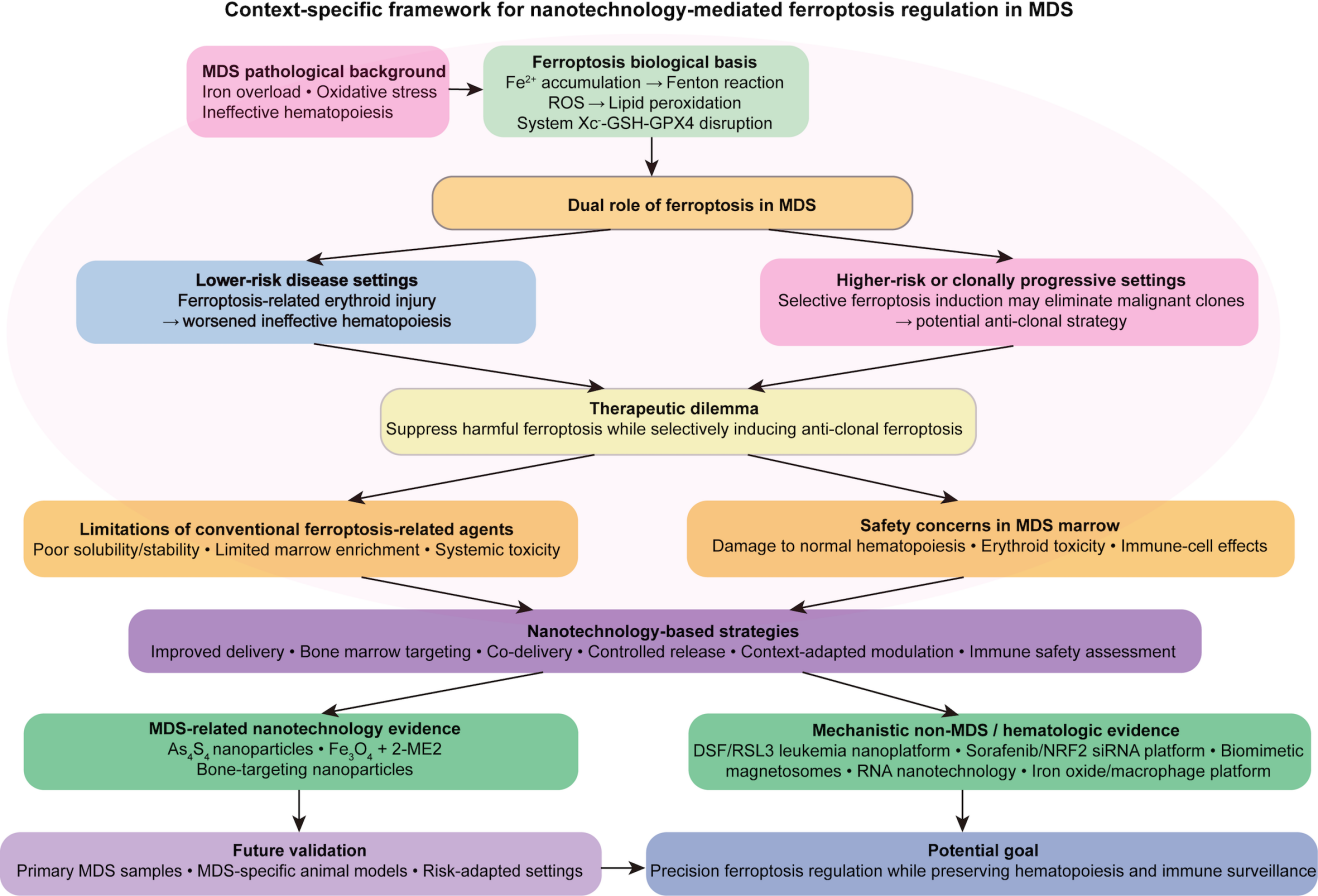
Context-specific framework for nanotechnology-mediated ferroptosis regulation in MDS. The image summarizes the pathological basis of ferroptosis in MDS, its dual role in lower-risk disease settings characterized by ineffective erythropoiesis and anemia versus in higher-risk or clonally progressive disease settings, and the therapeutic dilemma of suppressing harmful ferroptosis while inducing beneficial ferroptosis. It further highlights how nanotechnology may facilitate precision ferroptosis regulation and outlines current evidence gaps and future validation priorities. MDS, myelodysplastic syndromes; ROS, reactive oxygen species; GSH, glutathione; GPX4, glutathione peroxidase 4; Nrf2, nuclear factor erythroid 2-related factor 2.

Within this disease-specific marrow context, the dual role of ferroptosis creates both therapeutic opportunities and safety challenges. A major challenge is to enhance anti-clonal therapeutic efficacy while preserving normal bone marrow hematopoiesis. Nanotechnology may provide a promising strategy to address these challenges (21). Nanodrug delivery systems offer several advantages over conventional drug delivery approaches, including improved drug stability, prolonged circulation time, enhanced bioavailability, and targeted delivery to disease-relevant tissues or cellular populations (22, 23). These properties may facilitate more selective ferroptosis modulation within the bone marrow microenvironment. Based on these considerations, this review summarizes the biological roles and therapeutic implications of ferroptosis in MDS, with particular emphasis on recent advances in nanotechnology-based precision modulation of ferroptosis. The context-specific framework of this review is illustrated in **Figure 1**.

Relevant studies published up to May 2026 were identified through PubMed, Web of Science, and other major databases. Search terms included “myelodysplastic syndrome”, “ferroptosis”, “iron overload”, “oxidative stress”, “nanotechnology”, “nanoparticles”, “drug delivery”, “bone marrow microenvironment”, and “bone marrow targeting”, used alone or in combination. The literature selection focused on ferroptosis, iron metabolism, oxidative stress, ferroptosis-associated genes, and nanotechnology-based therapeutic approaches in MDS. Because direct evidence on nanotechnology-mediated ferroptosis regulation in MDS remains limited, relevant studies from other hematologic malignancies and solid tumors were included to provided mechanistic insights.

## 2 Ferroptosis in MDS

### 2.1 Core mechanisms of ferroptosis

Ferroptosis is an iron-dependent form of regulated cell death that is distinct from apoptosis and pyroptosis. Its hallmark is the accumulation of iron-dependent lipid peroxides, which ultimately results in membrane damage and cell death (12). Under normal conditions, cells rely on antioxidant systems, particularly GPX4, to eliminate lipid peroxides and maintain membrane stability. When antioxidant defenses are impaired, lipid peroxides accumulate continuously in cellular membranes, eventually leading to membrane damage and cell death. Ferroptosis is therefore considered a complex process driven by dysregulation of iron metabolism, lipid metabolism, and antioxidant pathways (24). Morphologically, ferroptotic cells typically show shrunken mitochondria, increased membrane density, reduced or absent mitochondrial cristae, and, in some cases, rupture of the outer mitochondrial membrane (12). The System Xc^-^–GSH–GPX4 axis is widely recognized as a key regulatory pathway in ferroptosis (12). System Xc^-^, a membrane transporter mainly composed of SLC7A11/xCT and SLC3A2, imports extracellular cystine into cells. Once inside the cell, cystine is reduced to cysteine, which is required for glutathione (GSH) synthesis. GPX4 uses GSH as a cofactor to convert toxic lipid hydroperoxides (LOOH) into non-toxic lipid alcohols (LOH), thereby preventing the propagation of lipid peroxidation. Therefore, GPX4 plays a central role in suppressing lipid peroxidation and protecting cells against ferroptosis.

**Figure 2:**
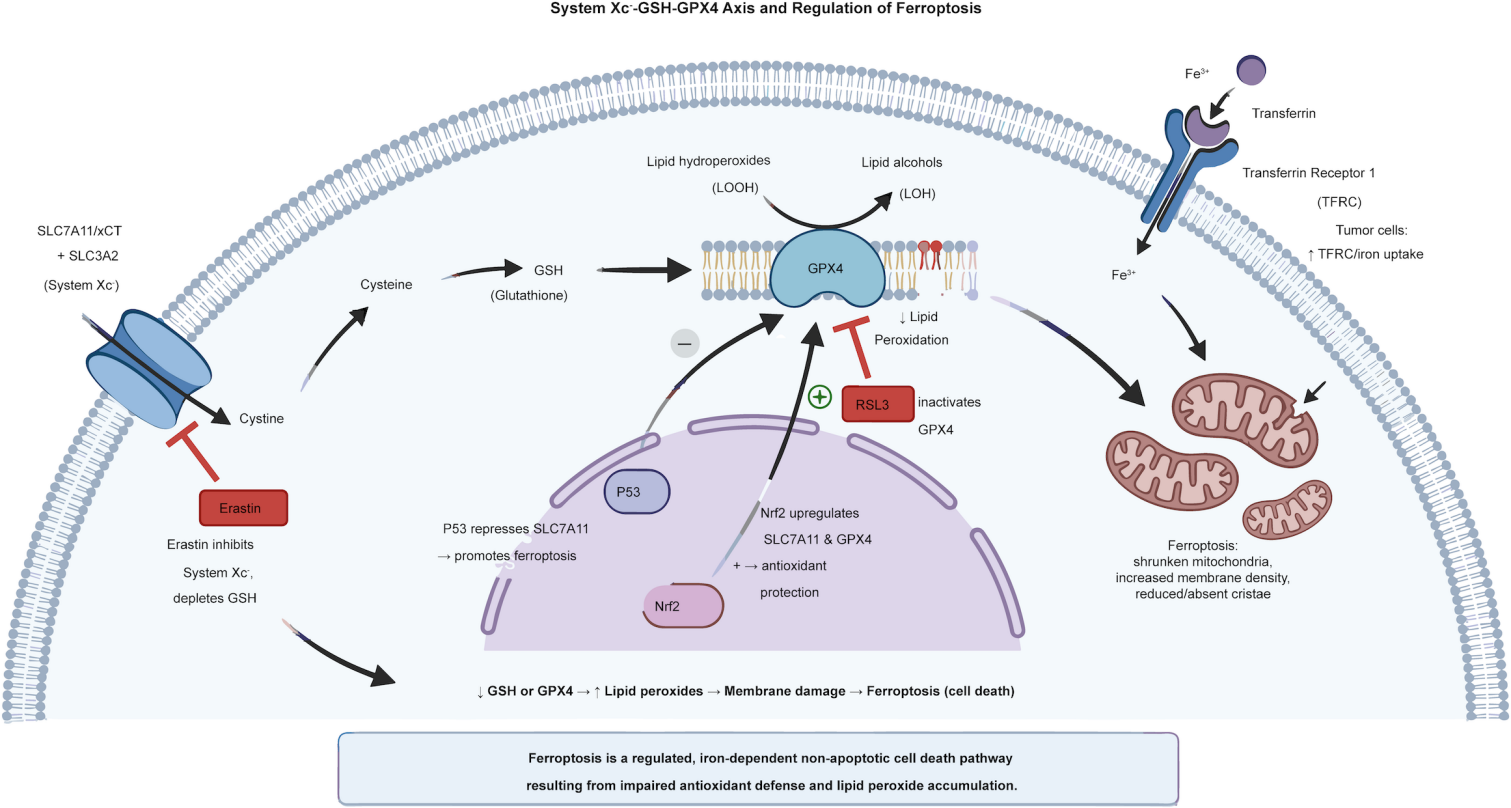
System Xc^-^–GSH–GPX4 axis and regulation of ferroptosis. The image summarizes the major molecular pathways regulating ferroptosis, including System Xc^-^, GSH synthesis, GPX4 activity, iron metabolism, and key regulatory factors involved in ferroptosis induction or suppression. GSH, glutathione; GPX4, glutathione peroxidase 4; RSL3, RAS-selective lethal 3; P53, tumor protein P53; SLC7A11/xCT, solute carrier family 7 member 11/cystine-glutamate transporter; TFRC, transferrin receptor 1; LOOH, lipid hydroperoxides; ROS, reactive oxygen species.

When the System Xc^-^–GSH–GPX4 axis is disrupted, intracellular GSH becomes depleted and GPX4 activity declines. As a result, lipid peroxides accumulate continuously and eventually trigger ferroptotic cell death. This pathway is tightly regulated by multiple signaling molecules. For example, P53 promotes ferroptosis by suppressing SLC7A11 transcription (25), whereas Nrf2 exerts antioxidant protection through upregulation of SLC7A11 and GPX4 expression (23). Several small molecules can also induce ferroptosis by targeting this pathway. Erastin mainly inhibits System Xc^-^ and depletes GSH, while RSL3 directly inactivates GPX4 (26–28). In addition, many tumor cells exhibit increased sensitivity to ferroptosis because of their high iron demand and reprogrammed iron metabolism, which is frequently accompanied by increased expression of transferrin receptor 1 (TFRC), enhanced iron uptake, and expansion of the intracellular labile iron pool (**Figure 2**).

### 2.2 Evidence of ferroptosis in MDS

Emerging evidence suggests that ferroptosis may influence both MDS pathogenesis and therapeutic response. Current evidence can be broadly categorized as clinical, experimental, and bioinformatic evidence, as summarized in **Table 1**. Most studies have not performed strict IPSS-R-defined subgroup analyses but have instead examined ferroptosis in specific biological or experimental contexts, including erythroid-lineage dysfunction, ineffective erythropoiesis, early disease-stage pathway alterations, iron-overload models, and MDS/AML models evaluating ferroptosis induction as a potential anti-clonal strategy (5, 17–20). Accordingly, the evidence discussed in this review is interpreted primarily according to biological and experimental context rather than formal IPSS-R risk stratification.

**Table 1:** Evidence synthesis of ferroptosis-related findings in MDS.

Evidence category	Study	Relevance to ferroptosis in MDS	Experimental model / data source	Major ferroptosis-related findings	Therapeutic or clinical implications	Limitations	Reference
Clinical evidence	Zhang et al., 2024	High	Bone marrow CD235a⁺ nucleated erythrocytes from MDS patients	Increased ferroptosis in MDS bone marrow nucleated erythrocytes, characterized by Fe²⁺ accumulation, lipid peroxidation, GSH depletion, MDA increase, and altered ACSL4/GPX4/SLC7A11 expression.	Ferroptosis of erythroid precursors may contribute to anemia and ineffective erythropoiesis in MDS.	Focused on erythroid-lineage cells; causal contribution to anemia and ineffective erythropoiesis requires further validation.	(17)
Clinical and mechanistic evidence	Yang et al., 2025	High	GlycoA⁺ nucleated erythrocytes from MDS patients; K562 and SKM1 cell models with FTH1 knockdown	FTH1 expression was reduced in MDS erythroid cells. FTH1 knockdown increased ferroptosis through ferritinophagy/autophagy induction and impaired erythroid differentiation and hemoglobin synthesis.	Supports FTH1-mediated ferritinophagy/ferroptosis as a mechanism contributing to MDS-associated anemia and a potential erythroid-directed therapeutic target.	Functional validation was mainly based on cell-line models; risk-stratified clinical validation and in vivo confirmation remain limited.	(5)
Clinical and translational evidence	Wu et al., 2025	Moderate	Retrospective clinical data from newly diagnosed MDS patients, GEO datasets, and NUP98-HOXD13 transgenic mouse model	Clinical data showed progressive ineffective erythropoiesis in MDS. Transcriptomic and mouse-model analyses showed that heme metabolism and ferroptosis pathways were upregulated in pre-disease and early stages; altered GPX4 and NCOA4 expression and TEM findings supported early ferroptosis involvement.	Suggests that ferroptosis-related pathways may contribute to early ineffective erythropoiesis and may represent stage-specific therapeutic targets in MDS-associated bone marrow failure.	Clinical data support ineffective erythropoiesis but do not directly prove ferroptosis. Ferroptosis was examined as part of broader pathway dynamics; ferroptosis-targeted intervention was not tested; the mouse model may not fully represent human MDS heterogeneity.	(18)
Experimental evidence	Lv et al., 2020	High	MDS patient-derived bone marrow mononuclear cells, SKM-1 and MUTZ-1 MDS cell lines, and C57BL/6 iron-overload mouse model.	MDS cells showed ferroptosis-related changes, including increased ROS accumulation, GSH depletion, and reduced GPX4 activity. Decitabine-induced cytotoxicity was partially reversed by ferroptosis inhibition or iron chelation, while erastin enhanced the cytotoxicity of decitabine.	Ferroptosis may contribute to the anti-clonal effects of hypomethylating agents and may represent a treatment-related cell death mechanism in MDS.	The mouse model was based on iron overload rather than a disease-specific MDS model; prospective clinical validation and in vivo therapeutic testing are lacking.	(19)
Experimental evidence	Li et al., 2024	High	CD33⁺ cells from MDS patients, MDS and AML cell lines	Erastin inhibited cell growth by inducing ferroptosis, accompanied by GSH depletion and GPX4 suppression. Combination with hypomethylating agents enhanced cytotoxicity.	Ferroptosis induction may enhance anti-clonal activity, particularly in higher-risk disease or HMA-based combination strategies.	Mainly in vitro; limited primary MDS functional validation; no in vivo or clinical validation.	(20)
Experimental evidence	Liu et al., 2024	Moderate	MDS-L and SKM-1 cell lines; primary bone marrow mononuclear cells from MDS patients; xenograft mouse model	Although RSL3 is commonly regarded as a GPX4 inhibitor and ferroptosis inducer, it suppressed MDS cell proliferation and BMMC viability mainly through ROS-dependent apoptosis via the MYB/Bcl-2 pathway rather than ferroptosis.	RSL3 demonstrated anti-MDS activity and showed synergistic effects with decitabine, suggesting potential therapeutic value and highlighting crosstalk between ferroptosis-associated compounds and other cell death pathways.	The study did not demonstrate ferroptosis as the primary mechanism of cell death; mechanistic conclusions were largely based on cell lines and require further validation in MDS subtypes and clinical samples.	(28)
Bioinformatic evidence	Zhu et al., 2024	Moderate	Public MDS datasets and qRT-PCR validation in clinical samples	A six-gene ferroptosis-related diagnostic signature, including SREBF1, PTPN6, PARP9, MAP3K11, MDM4, and EZH2, was identified and showed high diagnostic accuracy for MDS.	Ferroptosis-related gene signatures may have diagnostic value and may provide clues to immune-related mechanisms in MDS.	Mainly bioinformatic and diagnostic; functional roles of the identified genes in MDS ferroptosis remain unclear.	(31)
Bioinformatic evidence	Chen et al., 2023	Moderate	Public transcriptomic datasets and qRT-PCR validation in MDS bone marrow samples	BNIP3, MDM2, and RRM2 were identified as ferroptosis-related genes with diagnostic and prognostic value in MDS.	These genes may serve as candidate biomarkers for risk stratification and provide clues for ferroptosis-related therapeutic exploration.	Mainly bioinformatic; direct mechanistic validation is lacking.	(32)

Relevance to ferroptosis in MDS was qualitatively classified as high or moderate. High relevance was assigned to studies that directly assessed ferroptosis-related endpoints in MDS-specific samples or models and provided mechanistic, cellular, or therapeutic evidence. Moderate relevance was assigned to studies providing supportive but indirect evidence, including pathway-level, bioinformatic, association-based, partially validated, or mixed-mechanism findings, without definitive evidence that ferroptosis was the primary causal mechanism.

AML, acute myeloid leukemia; ACSL4, acyl-CoA synthetase long-chain family member 4; BMMC, bone marrow mononuclear cell; GEO, Gene Expression Omnibus; GSH, glutathione; GPX4, glutathione peroxidase 4; HMA, hypomethylating agent; MDA, malondialdehyde; MDS, myelodysplastic syndromes; MDS-L, myelodysplastic syndrome-leukemia cell line; NCOA4, nuclear receptor coactivator 4; ROS, reactive oxygen species; SLC7A11, solute carrier family 7 member 11; TEM, transmission electron microscopy.

Studies using AML or mixed MDS/AML models were considered supportive rather than definitive MDS-specific evidence.

#### 2.2.1 Clinical evidence

Chronic iron overload is a common feature of MDS and contributes substantially to disease progression. Excess iron can promote the generation of reactive oxygen species (ROS) through Fenton chemistry, leading to persistent oxidative stress within the bone marrow microenvironment. Given the close relationship among iron metabolism, oxidative stress, and ferroptosis, increasing attention has been directed toward the potential role of ferroptosis in the pathogenesis of MDS (16, 29).

Recent studies based on patient samples have provided additional support for the involvement of ferroptosis in MDS. Zhang et al. observed increased intracellular iron accumulation and lipid peroxidation in erythroid cells from patients with MDS compared with healthy controls, suggesting a possible contribution of ferroptosis to ineffective erythropoiesis and anemia (17). In another recent study, Yang et al. found reduced FTH1 expression in GlycoA^+^ nucleated erythrocytes from patients with MDS. Knockdown of FTH1 in MDS cells increased ferroptosis through ferritinophagy/autophagy induction and impaired erythroid differentiation and hemoglobin synthesis (5). In a related context, Wu et al. highlighted the interplay among oxidative stress, ineffective erythropoiesis, and abnormal signaling pathways in MDS (18). While these findings do not establish a direct causal role for ferroptosis, they underscore the need for further investigation into its contribution to MDS development and progression.

#### 2.2.2 Experimental evidence

Experimental studies have provided additional mechanistic insight into ferroptosis regulation in MDS. Lv et al. systematically investigated ferroptosis in MDS and found that MDS cells displayed enhanced ROS accumulation, glutathione depletion, and reduced GPX4 activity, all of which are consistent with ferroptotic vulnerability (19). Notably, decitabine-induced cytotoxicity was partially reversed by ferroptosis inhibitors, suggesting that ferroptosis may contribute to the therapeutic effects of hypomethylating agents. This study also indicated that distinct forms of regulated cell death may predominate in different disease-risk contexts, underscoring the complexity of ferroptosis regulation during disease progression.

Similarly, Li et al. reported that the ferroptosis inducer erastin inhibited the growth of MDS/AML cell models by reducing glutathione levels and suppressing GPX4 activity (20). Nevertheless, direct validation in primary MDS samples and *in vivo* MDS models remains limited, and these findings should therefore be interpreted with caution. Additional indirect support comes from studies of ferroptosis-associated hematopoietic injury. Bao et al. demonstrated that benzene exposure induced myelodysplasia-like hematotoxicity in mice. Importantly, ferroptosis inhibitors partially restored hematopoietic function. Although this model does not represent primary MDS, it provides mechanistic evidence that ferroptosis-mediated injury may contribute to hematopoietic dysfunction and supports further investigation of ferroptosis in MDS (30).

#### 2.2.3 Bioinformatic evidence

Bioinformatic analyses have also suggested a potential association between ferroptosis-related genes and MDS diagnosis, prognosis, and immune regulation. Zhu et al. constructed and experimentally validated a ferroptosis-related gene signature for MDS, including SREBF1, PTPN6, PARP9, MAP3K11, MDM4, and EZH2 (31). This model showed diagnostic potential and suggested that ferroptosis-related molecular networks may be involved in MDS pathobiology. Chen et al. further identified ferroptosis-related genes with potential diagnostic and prognostic value in patients with myelodysplastic neoplasms, including BNIP3, MDM2, and RRM2 (32). These genes are involved in processes such as oxidative stress regulation, mitochondrial function, DNA damage response, and iron-related cell death pathways. In addition, RAG1 has been identified as a ferroptosis-related gene in the FerrDb database (https://zhounan.org/ferrdb/current/). Huang et al. found that low RAG1 expression was associated with poor long-term survival in patients with MDS (33). These observations suggest a potential association between ferroptosis-related molecular alterations and disease prognosis in MDS, warranting further investigation. Many gene signatures require further validation in independent cohorts, primary patient samples, and functional experimental models. Therefore, bioinformatic evidence should be viewed as hypothesis-generating rather than definitive proof of ferroptosis involvement in MDS.

Overall, as summarized in **Table 1**, evidence supporting the involvement of ferroptosis in MDS remains heterogeneous and incomplete. Clinical studies primarily indicate associations among iron overload, oxidative stress, ineffective erythropoiesis, and ferroptosis-related cellular injury, whereas experimental studies provide the strongest mechanistic support. In contrast, most bioinformatic studies remain exploratory and mainly identify ferroptosis-related genes with potential diagnostic or prognostic value. Current evidence is further limited by small sample sizes, the lack of robust MDS-specific animal models, limited risk-stratified analyses, and insufficient distinction between effects on malignant clones and normal hematopoietic cells. These limitations highlight the need for nanotechnology-based platforms capable of enabling cell-selective ferroptosis modulation and *in vivo* validation within the bone marrow microenvironment.

### 2.3 Dual roles of ferroptosis in MDS

Studies suggest that ferroptosis plays a dual and context-dependent role in MDS. In lower-risk disease settings characterized by ineffective erythropoiesis and anemia, the bone marrow microenvironment is often exposed to oxidative stress driven by ineffective erythropoiesis and iron overload. Excess free iron promotes ROS generation through the Fenton reaction, leading to lipid peroxidation and potentially triggering ferroptosis-related injury in erythroid progenitor or erythroid-lineage cells. This process may further aggravate ineffective hematopoiesis and anemia.

In higher-risk or clonally progressive disease settings, selective induction of ferroptosis in malignant clonal cells may represent a potential therapeutic strategy. Previous studies have shown that hypomethylating agents (HMAs) may exert part of their antitumor effects through ferroptosis induction. Lv et al. (19) reported that decitabine treatment increased ROS levels, depleted GSH, and reduced GPX4 activity in MDS cells. These effects were partially reversed by the ferroptosis inhibitor Ferrostatin-1 (Fer-1) and the iron chelator deferoxamine (DFO), indicating that ferroptosis may play an important role in decitabine-induced cell death. In addition, combining erastin with decitabine or azacitidine further decreased GPX4 activity and enhanced cytotoxicity in MDS cells, indicating that ferroptosis induction may improve the therapeutic efficacy of HMAs (19, 20). Eprenetapopt (APR-246), a p53-reactivating agent with reported ferroptosis-inducing activity, showed promising clinical activity when combined with azacitidine in TP53-mutant MDS/AML (34, 35).

These findings highlight a central challenge in ferroptosis regulation in MDS: depending on disease context, therapeutic strategies may require either suppression of ferroptosis to preserve erythropoiesis and normal hematopoiesis or induction of ferroptosis to eliminate malignant clones. Iron chelation therapy exemplifies this therapeutic complexity. By reducing intracellular iron availability, iron chelators may alleviate oxidative stress and ferroptosis-related injury, which could be beneficial in disease settings characterized by iron overload, ineffective erythropoiesis, and anemia. However, reduced iron availability may also decrease ROS accumulation and lipid peroxidation, thereby weakening ferroptosis-dependent antitumor effects. For example, in hepatocellular carcinoma models, deferasirox (DFX) was shown to inhibit sorafenib-induced ferroptosis, although it retained antitumor activity through additional mechanisms, including NF-κB inhibition and the induction of apoptosis and necroptosis (36). Complementary findings from AML suggest that certain drug-resistant malignant cells may acquire new ferroptosis vulnerabilities. Venetoclax-resistant AML cells remain sensitive to ferroptosis inducers, indicating that metabolically reprogrammed malignant cells may become more dependent on iron metabolism and redox balance (37). These findings raise the possibility that resistant clones in higher-risk or clonally progressive MDS may also remain susceptible to ferroptosis induction. Collectively, current evidence suggests that both ferroptosis suppression and ferroptosis induction may have therapeutic relevance in MDS. Future strategies will therefore require context-specific and cell-selective modulation of ferroptosis according to disease context, cellular target, and therapeutic objective.

### 2.4 Ferroptosis-related genes as candidate therapeutic targets

As summarized in **Table 1** and Section 2.2.3, several ferroptosis-related genes or gene signatures have been associated with MDS diagnosis, prognosis, or immune-related features (31, 32, 38). However, their therapeutic significance remains largely hypothetical because most available evidence comes from bioinformatic analyses, retrospective datasets, or limited validation of clinical samples. Direct functional evidence demonstrating that these genes regulate ferroptosis in MDS cells remains insufficient (31–33). Therefore, these genes should currently be regarded as candidate biomarkers or hypothesis-generating targets rather than validated therapeutic targets.

If functionally validated ferroptosis-related targets are identified, their therapeutic application will require context-adapted and cell-selective modulation, given the distinct effects of ferroptosis in erythroid-lineage cells, malignant MDS cells, residual normal hematopoietic compartments, and immune cell populations (17, 19, 20). Nanotechnology-based delivery systems may help achieve such selective modulation by leveraging their inherent advantages (39).

## 3 Nanotechnology-mediated ferroptosis in MDS: current progress and future perspectives

Research at the intersection of nanotechnology, ferroptosis, and MDS is still at an early stage of development. Most nanotechnology-based studies in MDS have focused on improving bone marrow-targeted drug delivery, reducing systemic toxicity, or correcting ineffective erythropoiesis, rather than directly targeting ferroptosis pathways (40). While direct MDS-specific evidence remains limited, studies from solid tumors and other hematologic malignancies have demonstrated the feasibility of nanotechnology-mediated ferroptosis regulation. Therefore, the evidence discussed in the following sections should be interpreted with caution. Studies from these systems are included primarily to provide mechanistic insights into nanotechnology-mediated ferroptosis regulation. As summarized in **Table 2**, currently available nanotechnology platforms include both MDS-related delivery systems and ferroptosis-oriented nanoplatforms developed in other malignancies, which are included here for their mechanistic relevance. The selected platforms represent key examples rather than an exhaustive collection of all available studies.

**Table 2:** Comparative summary of MDS-related and mechanistically relevant nanotechnology platforms for ferroptosis-based therapeutic strategies.

Category	Nanotechnology platform	Experimental model / evidence source	Main mechanism	Potential advantages	Major limitations	Potential relevance to MDS	Translational potential in MDS	Reference
MDS-related platform	Hydrophilic polymer-coated As₄S₄ nanoparticles	MDS patient-derived bone marrow mononuclear cells	Promoted terminal erythropoiesis, increased globin expression and CD235a-positive erythroid differentiation, induced eIF2α phosphorylation, and reduced intracellular ROS	MDS-related evidence; may improve ineffective erythropoiesis and oxidative stress	Direct ferroptosis endpoints were not evaluated; relationship with ferroptosis remains indirect	May be relevant for lower-risk MDS with anemia and ineffective erythropoiesis	Moderate	(40)
MDS-related platform	Magnetic Fe₃O₄ nanoparticles combined with 2-ME2	SKM-1 MDS cell line	Enhanced inhibitory effects on MDS cells; mainly assessed cell-cycle progression and apoptosis	MDS cell-line evidence; may improve drug delivery	Ferroptosis-specific markers were not assessed; no in vivo MDS validation	Supportive evidence for nanotechnology application in MDS cells, but not direct nano-ferroptosis evidence	Moderate	(47)
MDS-related platform	Bone-targeting nanoparticles	MDS mouse model	Enhanced bone-targeted drug accumulation, improved therapeutic efficacy, and reduced systemic toxicity	MDS-specific in vivo evidence; supports bone-targeted co-delivery	Ferroptosis-specific endpoints were not examined	Supports bone-targeted delivery strategies for MDS treatment; not direct nano-ferroptosis evidence	High	(48)
Hematologic malignancy-related nano-ferroptosis platform	Leukemia cell membrane-coated multi-metal nanoplatform loaded with DSF/RSL3	AML cell and animal models	pH-responsive release of copper, iron, manganese ions, DSF, and RSL3; Fenton-like ROS generation, GSH depletion, ferroptosis/cuproptosis induction, and cGAS-STING-mediated immune activation.	Hematologic malignancy-related evidence; combines ferroptosis, cuproptosis, and immune activation; may improve leukemia-cell targeting	AML rather than MDS model; effects on normal hematopoiesis and MDS bone marrow immunity remain unknown	Exploratory framework for higher-risk MDS strategies combining ferroptosis induction, redox modulation, and immune activation	Exploratory	(52)
Mechanistic nano-ferroptosis platform	Hyperbranched polymer nanocarrier	Hepatocellular carcinoma model (animal experiments)	Improved sorafenib delivery and inhibited NRF2 antioxidant defense to enhance ferroptosis	Demonstrates co-delivery strategy targeting ferroptosis resistance	Solid tumor evidence; not validated in clinical samples	Mechanistic rationale for future MDS-focused co-delivery strategies	Exploratory	(42)
Mechanistic nano-ferroptosis platform	Biomimetic leukocyte membrane-coated magnetosomes	Solid tumor mouse models	Leukocyte membrane-coated Fe₃O₄ magnetosomes co-delivered PD-1 antibody and TGF-β inhibitor, promoted immunomodulation, enhanced Fenton reaction-mediated ROS generation, and induced tumor ferroptosis.	Prolonged circulation, reduced immune clearance, MRI-guided magnetic targeting, and enabled combined ferroptosis-immunomodulation therapy.	Non-MDS evidence; immune effects in MDS bone marrow remain unknown	Exploratory strategy for immune-compatible and image-guided delivery; MDS-specific validation is required.	Exploratory	(43)
Gene-regulatory nanoplatform	RNA nanotechnology platforms	Cancer immunotherapy models / review	Protect therapeutic RNAs from degradation and enable gene regulation by delivering siRNA, microRNA, mRNA, or CRISPR-related components to tumor or immune cells.	May enable programmable gene regulation and immune modulation; potential application to ferroptosis-related targets remains exploratory.	No direct MDS ferroptosis evidence; delivery specificity and safety require validation	Potential future approach for regulating ferroptosis- or immune-related genes in MDS; requires MDS-specific validation.	Exploratory	(44)
Immune-modulating nanoplatform	Iron oxide nanoparticle-based macrophage-modulating platform	Solid tumor mouse model	Promotes M1 macrophage polarization and Fenton reaction-related ROS production, leading to tumor cell apoptosis.	Promotes M1 macrophage polarization and antitumor effects.	Non-MDS evidence; not ferroptosis-specific; uncertain safety in iron-overloaded MDS marrow.	Mechanistic support for macrophage-oriented nanomodulation; MDS-specific validation is required	Exploratory	(51)

Translational potential in MDS was classified as high, moderate, or exploratory. High indicates MDS-specific in vivo evidence or strong disease-relevant delivery evidence; moderate indicates MDS-related patient-sample or cell-line evidence with limited validation or indirect ferroptosis relevance; exploratory indicates non-MDS or conceptual evidence requiring MDS-specific validation.

AML, acute myeloid leukemia; As₄S₄, arsenic sulfide; cGAS-STING, cyclic GMP-AMP synthase–stimulator of interferon genes; CRISPR, clustered regularly interspaced short palindromic repeats; DSF, disulfiram; eIF2α, eukaryotic translation initiation factor 2 alpha; FDA, Food and Drug Administration; GPX4, glutathione peroxidase 4; GSH, glutathione; MDS, myelodysplastic syndromes; MRI, magnetic resonance imaging; NRF2, nuclear factor erythroid 2-related factor 2; PD-1, programmed cell death protein 1; ROS, reactive oxygen species; siRNA, small interfering RNA; TGF-β, transforming growth factor-β; 2ME2, 2-methoxyestradiol.

### 3.1 Nanotechnology improves delivery efficiency of ferroptosis-related therapeutics

Ferroptosis-related agents, including erastin, RSL3, sorafenib, and several natural bioactive compounds, often face limitations in clinical applications, such as poor stability, short circulation time, limited accumulation at disease sites, and systemic toxicity (41). Nanodrug delivery systems (NDDSs) have therefore attracted increasing attention as a potential strategy to improve ferroptosis-based therapy.

At present, a variety of nanoplatforms have been developed to improve the delivery of ferroptosis inducers. For example, an amphiphilic hyperbranched polyglycerol (HDP) nanosystem co-loaded with sorafenib and Nrf2 siRNA was reported to improve drug solubility and further enhance ferroptosis induction (42). Biomimetic approaches, such as leukocyte membrane-coated nanoparticles, have been shown to prolong circulation time and improve therapeutic delivery (43). In addition, RNA nanotechnology offers a potential platform for targeted modulation of ferroptosis-related pathways through the delivery of therapeutic nucleic acids (44, 45). Importantly, these nanoplatforms were developed mainly in solid tumor models.

### 3.2 Bone marrow-targeted nanoplatforms for MDS therapy

Bone marrow targeting is particularly relevant to MDS because the disease originates from clonal hematopoietic stem and progenitor cells within the marrow niche. Unlike solid tumors, MDS does not form a discrete tumor mass, making targeted drug delivery more challenging. Therapeutic agents must be distributed within the bone marrow microenvironment while avoiding excessive exposure to normal hematopoietic cells. Therefore, improving delivery specificity within the bone marrow microenvironment remains a key objective for nanotechnology-based therapies in MDS.

In recent years, increasing attention has been paid to nanotechnology in bone and bone marrow–related diseases. Magnetic nanoparticles may improve drug delivery efficiency because of their high surface area-to-volume ratio and cellular uptake properties (46). In MDS, the combination of 2-methoxyestradiol (2-ME2) and Fe_3_O_4_ magnetic nanoparticles was reported to enhance inhibitory effects on SKM-1 cells (47). In addition, bone-targeting nanoparticles (BTNPs) co-delivering decitabine and arsenic trioxide enhanced drug accumulation within the bone marrow, improved therapeutic efficacy, and reduced systemic toxicity in an MDS mouse model (48).

However, these studies did not directly assess ferroptosis-specific endpoints, such as lipid peroxidation, GPX4/GSH activity, ferroptosis inhibitors, or rescue experiments. Consequently, current MDS-specific nanotechnology studies support the feasibility of bone marrow-targeted drug delivery but do not yet provide direct evidence for ferroptosis-based therapeutic effects.

### 3.3 Ferroptosis-inducing nanomaterials: mechanistic promise and evidence gaps

Several nanotechnology platforms have been investigated in MDS, including hydrophilic polymer-coated As_4_S_4_ nanoparticles, Fe_3_O_4_ magnetic nanoparticles combined with 2-methoxyestradiol (2-ME_2_), and bone-targeting nanoparticles (**Table 2**). Although these platforms were not designed to directly induce ferroptosis, studies in other malignancies have shown that nanomaterials can modulate ferroptosis through ROS amplification, disruption of the GSH–GPX4 axis, or co-delivery of ferroptosis-regulating agents (49–52). Nevertheless, direct extrapolation from solid tumors to MDS should be approached with caution. Ferroptosis appears to exert context-dependent effects in MDS (17, 19, 20). Non-selective activation of ferroptosis within the bone marrow could produce unintended detrimental effects.

This concern is particularly relevant to iron-based nanomaterials. Although iron oxide and other iron-containing nanoparticles have been shown to enhance ROS generation and lipid peroxidation in tumor models (53, 54), many patients with MDS already exhibit systemic or bone marrow iron overload. Future studies should therefore determine whether these nanoplatforms can selectively induce ferroptosis in malignant clones without exacerbating erythroid injury or impairing residual hematopoiesis.

### 3.4 Nanotechnology for dual role ferroptosis regulation in MDS

The ferroptosis paradox in MDS reflects the context-dependent roles of ferroptosis during disease evolution. Ferroptosis-related pathways may contribute to ineffective erythropoiesis and anemia during the early stages of disease development (18), whereas selective induction of ferroptosis in malignant clones may be therapeutically beneficial in higher-risk or clonally progressive disease settings. Consequently, ferroptosis modulation in MDS may require either inhibition or induction of ferroptosis depending on the biological and clinical context. These contrasting requirements highlight the need for nanotechnology-based approaches capable of context-specific ferroptosis modulation.

Because nanoplatforms can improve drug delivery specificity and cellular targeting, they may provide a means to achieve such selective ferroptosis regulation. Studies in leukemia have suggested that nano-formulations may enhance the antileukemic activity of ferroptosis inducers while reducing off-target toxicity to normal hematopoietic cells (55). However, whether a similar strategy can achieve context-specific ferroptosis modulation in MDS remains unknown.

### 3.5 Immune-cell-specific considerations for nanotechnology-mediated ferroptosis strategies in MDS

Ferroptosis can influence multiple aspects of immune-cell function, including cell survival, cytokine production, antigen presentation, inflammatory signaling, and antitumor immune surveillance (56–58). Because immune dysregulation is a hallmark of MDS, the effects of ferroptosis-modulating nanoplatforms on immune cells warrant careful consideration. Non-selective ferroptosis induction may compromise protective immune functions, highlighting the importance of immune-cell-specific evaluation during nanoplatform development.

#### 3.5.1 T and B lymphocytes

In adaptive immunity, T cells exemplify the dual role of ferroptosis in antitumor immunity. Activated CD8^+^ T cells can promote ferroptosis in tumor cells through IFN-γ-mediated signaling, which activates the JAK–STAT1 pathway, downregulates SLC7A11 and SLC3A2, impairs cystine uptake, and leads to lipid peroxidation (56). However, CD8^+^ T cells themselves may also undergo ferroptosis. Increased expression of the fatty acid transporter CD36 within the tumor microenvironment enhances lipid uptake and peroxidation, thereby increasing ferroptosis susceptibility and impairing antitumor function (59). B-cell subsets also exhibit differential sensitivity to ferroptosis. B1 and marginal zone B cells are highly dependent on GPX4-mediated protection against lipid peroxidation, whereas follicular B2 cells show relatively lower dependence (60). These findings suggest that different lymphocyte subsets may respond differently to modulation of ferroptosis, highlighting the importance of considering adaptive immune-cell responses during the development of ferroptosis-oriented nanoplatforms.

Notably, CD8^+^ T-cell-targeted nanoparticles loaded with the ferroptosis inhibitor ferrostatin-1 have demonstrated the feasibility of protecting specific lymphocyte populations from ferroptosis-associated injury in an inflammatory disease model (61). Although this approach has not been evaluated in MDS, it provides proof-of-concept that nanoplatforms can achieve cell-selective ferroptosis modulation.

#### 3.5.2 Macrophages

Macrophages play key roles in erythrophagocytosis, iron recycling, erythroid support, and inflammatory regulation within the bone marrow microenvironment (58, 62). Experimental studies have shown that excessive erythrophagocytosis can induce macrophage ferroptosis. In a murine transfusion model, the rapid uptake of large numbers of erythrocytes exceeded the protective capacity of heme oxygenase-1 (HO-1), ultimately triggering ferroptotic cell death in macrophages (63). Given the frequent occurrence of chronic transfusion dependence and iron overload in MDS, abnormal iron metabolism may increase macrophage susceptibility to ferroptosis through enhanced lipid peroxidation.

Macrophages are major phagocytic cells involved in nanoparticle uptake and biodistribution, making them both potential nanoparticle sinks and targets for nanoplatform-based modulation (64). In MDS, nanoparticle accumulation in macrophages may affect iron recycling and bone marrow homeostasis. In addition, macrophage ferroptosis can be actively regulated by nanoplatforms; for example, CD16/32-modified ZIF90 nanoparticles loaded with spermine inhibited macrophage ferroptosis through mitochondrial protection and upregulation of GPX4/xCT in an atherosclerosis model (65). Therefore, macrophage uptake, ferroptosis susceptibility, and iron-handling functions should be carefully evaluated when developing ferroptosis-oriented nanoplatforms for MDS.

#### 3.5.3 Dendritic cells

Dendritic cells (DCs) are key antigen-presenting cells that link innate and adaptive immunity and play a central role in initiating tumor-specific T-cell responses (66). Several studies have shown that ferroptosis induced by peroxisome proliferator-activated receptor gamma (PPARγ) activation, PD-L1 deficiency, or X-box binding protein 1 (XBP1)-driven lipid accumulation can impair antigen presentation, suppress CD8^+^ T-cell responses, and promote immune evasion (57, 67).

These observations suggest that the impact of ferroptosis-oriented nanoplatforms on dendritic-cell function should be considered during platform design for MDS. For example, a biomimetic Fe_3_O_4_-siPD-L1 nanoplatform induced tumor-cell ferroptosis while promoting dendritic-cell maturation and T-cell activation in a glioblastoma model (66), indicating that ferroptosis induction and immune activation may be integrated within a single nanoplatform design.

#### 3.5.4 Neutrophils

Emerging evidence suggests that tumor-infiltrating neutrophils (TINs) may exhibit context-dependent susceptibility to ferroptosis within the tumor microenvironment (57, 58). In gastric cancer, TINs undergo spontaneous ferroptosis, release oxidized lipids, and suppress T-cell activity (68). In contrast, TINs in breast cancer appear resistant to ferroptosis, partly because TIN-derived itaconic acid inhibits lipid peroxidation and promotes neutrophil survival (69). Together, these findings suggest that local metabolic and microenvironmental cues may shape neutrophil susceptibility to ferroptosis and subsequent immunoregulatory functions.

These characteristics may also influence the behavior of ferroptosis-oriented nanoplatforms within inflammatory microenvironments. For example, a neutrophil membrane-biomimetic liposome loaded with ferrostatin-1 was shown to target atherosclerotic plaques and reduce ROS-mediated ferroptotic injury (70, 71). Such findings illustrate the potential of neutrophil-associated nanoplatforms to modulate ferroptosis while exploiting innate immune-cell targeting properties.

#### 3.5.5 Natural killer cells

Natural killer (NK) cells contribute to antitumor immune surveillance and have emerged as potential immunotherapeutic targets in MDS. Nevertheless, NK-cell function is frequently impaired by the MDS immune microenvironment, and current NK-cell-based therapeutic approaches remain at an early stage of clinical development. Early clinical studies of NK-cell-based therapies in high-risk MDS and related myeloid malignancies have demonstrated acceptable safety, although therapeutic efficacy remains variable and requires further optimization (72). Preclinical studies have suggested that combining ferroptosis inducers with NK-cell-based immunotherapies may produce synergistic antitumor effects (73). Excessive lipid peroxidation, however, may impair NK-cell viability and cytotoxic activity, indicating that NK cells may also be vulnerable to ferroptosis-associated damage. Restoration of antioxidant defenses has been reported to improve NK-cell activity, suggesting that preservation of NK-cell function should be considered when designing nanotechnology-mediated ferroptosis strategies (74, 75).

Moreover, NK-cell-derived nanovesicle platforms loaded with ferroptosis-related agents, such as sorafenib, have demonstrated enhanced antitumor activity in non-MDS cancer models, suggesting the potential to combine ferroptosis induction with immune-mediated antitumor effects (76). These findings support further exploration of NK-cell-oriented ferroptosis nanoplatforms in MDS.

In summary, ferroptosis exerts diverse effects on multiple immune-cell populations. These effects may be beneficial or detrimental depending on the specific immune-cell population and disease context. Emerging nanotechnology-based platforms provide opportunities for cell-selective modulation of ferroptosis, enabling either the induction or inhibition of ferroptosis in specific target cells. Such strategies may facilitate cell-selective ferroptosis modulation in MDS, enabling the preferential targeting of malignant clones while preserving normal hematopoiesis and immune surveillance within the bone marrow microenvironment.

## 4 Challenges and future perspectives

Ferroptosis may participate in disease progression, ineffective hematopoiesis, and treatment responses in MDS. However, its role appears to be highly context dependent, and direct evidence remains limited, particularly for nanotechnology-mediated ferroptosis modulation.

### 4.1 Future research priorities

Future research should further clarify the effects of ferroptosis on malignant clones, residual normal hematopoietic cells, erythroid-lineage cells, and immune-cell populations within the bone marrow microenvironment (18, 20). Particular attention should be given to achieving context-specific and cell-selective ferroptosis modulation, distinguishing ferroptosis-related injury to normal hematopoiesis from therapeutically beneficial ferroptosis induction in malignant clones. More representative MDS-specific experimental systems, including patient-derived models and disease-relevant animal models, are needed to evaluate nanotechnology-mediated ferroptosis regulation under physiologically relevant conditions. Predictive biomarkers related to iron metabolism, lipid peroxidation, ferroptosis-regulatory pathways, and immune microenvironmental features should also be explored to support patient stratification and guide the application of nanotechnology-mediated, context-specific ferroptosis modulation strategies.

### 4.2 Translational challenges

A major translational challenge for ferroptosis-based nanotherapies in MDS is achieving selective targeting of malignant clones while preserving residual normal hematopoiesis and immune surveillance. This issue may be particularly relevant in patients with transfusion dependence and iron overload, where alterations in iron metabolism could further influence ferroptosis susceptibility (48, 77).

### 4.3 Clinical development directions

From a clinical development perspective, future nanotechnology-mediated ferroptosis strategies may be most valuable when integrated with existing MDS therapies rather than developed as isolated interventions. Potential directions include co-delivery systems combining ferroptosis inducers with hypomethylating agents, rational integration of ferroptosis modulation with iron chelation strategies, and multifunctional nanoplatforms capable of simultaneously modulating ferroptosis and immune responses within the bone marrow microenvironment. Ultimately, the development of nanoplatforms capable of stage-adapted and cell-selective ferroptosis modulation may provide a promising precision-medicine strategy for patients with MDS.

## 5 Conclusion

Ferroptosis appears to play context-dependent roles in MDS and serves as an important mechanistic link among iron overload, oxidative stress, ineffective hematopoiesis, and immune microenvironmental dysregulation. Nanotechnology offers a promising strategy for selective and controllable ferroptosis modulation, although direct MDS-specific evidence remains limited. Future progress will require MDS-specific validation of nanoplatforms and the development of bone marrow-oriented delivery systems capable of enhancing anti-clonal activity while preserving hematopoietic and immune homeostasis.
